# The effect of surface treatment with Er: YAG laser on shear bond strength of orthodontic brackets to fiber-reinforced composite

**DOI:** 10.4317/jced.51613

**Published:** 2014-10-01

**Authors:** Mahboobe Dehghani, Farzaneh Ahrari

**Affiliations:** 1 DDS MS, Assistant Professor of Orthodontics. Dental Research Center, School of Dentistry, Mashhad University of Medical Sciences, Mashhad, Iran

## Abstract

Objectives: This study aimed to investigate the effect of surface treatment with Er:YAG laser on shear bond strength (SBS) of orthodontic brackets to fiber-reinforced composite (FRC). Study Design: Ninety human premolars were randomly divided into six groups of 15. FRC bars were bonded to the teeth with a flowable composite (FC) and then underwent following treatments. In group 1 no further treatment was performed. In group 2 the FRC surfaces were covered by FC. An Er:YAG laser was employed to treat FRCs in groups 3 ( 200 mJ/10 Hz) and 4 (300 mJ/15 Hz). The FRC strips in groups 5 and 6 were first covered by FC and then irradiated with Er:YAG laser at 200 mJ/10 Hz (group 5) or 300 mJ/15 Hz (group 6). Stainless steel brackets were bonded to FRCs using a light-cure adhesive system. After 24 hours, the samples were tested for SBS and the adhesive remnant index (ARI) scores were determined. Results: There was a significant difference in SBS among the study groups (P <0.001). Pairwise comparisons indicated that SBS was significantly lower in group 1 compared to all other groups (p<0.05) except group 2. Bond strength in group 6 was significantly greater than all the study groups (p<0.05) except group 5. No significant difference was found in ARI scores among the groups. Conclusions: Covering the FRC surface by a layer of flowable composite and then application of Er:YAG laser at 300 mJ/15 Hz could be recommended to increase bond strength of orthodontic attachments to FRC.

** Key words:**Fiber-reinforced composite, orthodontics, Sshear bond strength, laser, Er:YAG, surface treatment, bracket, FRC.

## Introduction

Fiber reinforced composite (FRC) is a combination of long continuous fibers and a bisphenol-a-glycidyl dimet-hacrylate (Bis-GMA) matrix, which provides optimal mechanical properties such as high flexural and fracture strength and adequate flexural modulus (1-2). FRC offers an esthetic metal-free alternative for many applications in dentistry including periodontal splints, endodontic posts and fixed prosthodontic appliances, and it can be employed for stabilizing traumatized teeth (3-5). FRC can also be used for retention, space maintenance, anchorage and active tooth movement during orthodontic treatment (6-8). From the orthodontic point of view, the great advantages of FRCs include easy and fast bonding technique with no need to laboratory work, biocompatibility, excellent mechanical properties, esthetics, and reducing the need for bands, brackets and wires (1,9).

When employed as an anchorage or a tooth movement unit, several teeth are connected to each other by a FRC bar to produce a single rigid segment, and then an orthodontic bracket, tube or hook is directly bonded to the FRC connecting bar (6). There are a few studies regarding the bond strength of orthodontic attachments to FRCs and the results of previous studies in this field are controversial.

Since the introduction of Er:YAG (erbium-doped: yttrium aluminum garnet) laser into dentistry in 1997, it has been employed for different purposes; one of them is surface preparation of dental materials and tooth tissues. Recent experiments investigated the efficacy of erbium family (Er:YAG and Er,Cr:YSGG) lasers for etching dentin and enamel (10-14), surface roughening of composite restorations to enhance bonding (15-16), and bracket base reconditioning (17-19). According to the authors’ knowledge, Er:YAG laser has not been employed to date for FRC surface treatment. Considering the effectiveness of erbium lasers in roughening the surface (20) and increasing micromechanical retention (17-18), it seems that laser conditioning can be employed as a way to enhance bond strength of orthodontic attachments to FRC bars. Therefore, this study aimed to investigate the effect of FRC surface treatment with Er:YAG laser on shear bond strength (SBS) and mode of bond failure of orthodontic brackets.

## Material and Methods

Ninety freshly extracted human upper premolars were collected and stored in distilled water at room temperature until required. The selected teeth were intact with no caries, enamel cracks, or other developmental defects. The teeth were randomly divided into six groups of 15 each.

The FRC bars (Quartz Splint Woven, RTD, France) used in this study were 0.4 mm in diameter and were cut into lengths of 4 mm by scissors. The glass fibers were pre-impregnated with a proprietary resin matrix from the manufacturer.

Before bonding, the facial surfaces of the teeth were cleansed using a non-fluoridated pumice slurry and rubber prophylactic cups, rinsed with water and air-dried. The treatment procedures in the study groups were as follows:

Group 1: The buccal enamel was etched by application of a 37 per cent phosphoric acid gel for 30 seconds, thoroughly rinsed with water and dried with an oil-free air source. A thin layer of bonding agent (Tetric N-Bond; Ivoclar Vivadent, Schaan, Liechtenstein) was later applied on the etched enamel and polymerized for 20 seconds by a halogen light-curing unit (Australis 7; Ivoclar Vivadent). Afterwards, a layer of flowable composite (Heliomolar Flow, Ivoclar Vivadent) was placed on the enamel surface. A FRC bar was then pressed on the flowable composite at the center of the crown and the excess resin was removed with an explorer. The specimen was cured with the Australis 7 unit at the light intensity of 700 mW/cm2 for 40 seconds (20 seconds from the mesial and 20 seconds from the distal margins).

Group 2: The teeth in group 2 were etched with 37% phosphoric acid gel for 30 seconds, rinsed with water and air-dried. A thin layer of bonding agent (Tetric N-Bond; Ivoclar Vivadent) was applied on the etched enamel, polymerized for 20 seconds and then covered by a layer of flowable composite (Heliomolar Flow, Ivoclar Viva-dent). Afterwards, a FRC bar was placed on the flowable composite at the center of the crown and the excess resin was removed. The FRC-composite combination was then cured for 5 seconds, followed by the application of another layer of flowable resin to completely cover the fiber. Final curing was performed with the Australis 7 unit at the intensity of 700 mW/cm2 for 40 seconds (20 seconds from the mesial and 20 seconds from the distal margins).

Group 3: In this group, the bonding procedure was performed similar to that described in group 1 and then an Er:YAG laser (wavelength 2940 nm; Smart 2940 D, Deka Laser, Firenze, Italy) was applied for FRC surface treatment. The laser was held manually and operated at 200 mJ of energy, very short pulse, and frequency of 10 Hz with the use of air and water spray. The beam was irradiated in a focused non-contact mode at a distance of 5 mm and perpendicular to the FRC surface. The bonding area was treated for 10 seconds using scanning movements.

Group 4: The bonding procedure and FRC treatment was similar to that described in group 3, except that the Er:YAG laser was employed at 300mJ of energy and pulse frequency of 15 Hz.

Group 5: After placing FRC on the tooth surface, the composite-FRC combination was cured for 5 seconds and then the FRC bar was thoroughly covered by a thin layer of flowable composite as described in group 2. After 40 seconds of light curing, the Er:YAG laser was employed for 10 seconds using very short pulse, 200 mJ of energy and frequency of 10 Hz.

Group 6: The bonding procedure and FRC surface treatment was similar to group 5, except that 300 mJ of energy and pulse frequency of 15 Hz were employed.

Stainless steel standard edgewise maxillary central incisor brackets (0.018-in slot, 3M Unitek, Monrovia, CA, USA) were used in all the experimental groups. The FRC surface was coated by a thin layer of Transbond XT primer (3M Unitek) and light-cured for 10 seconds. Then the bracket base was covered by Transbond XT ad-hesive and firmly pressed to the FRC surface. The excess adhesive was removed from the periphery of the base with an explorer and light-polymerization was performed with the curing unit for 40 seconds (20 seconds from the mesial side and 20 seconds from the distal side of the bracket base).

After bonding, the specimens were stored in distilled water for 24 h at room temperature. Then each tooth was embedded in self-curing acrylic resin using a custom-made mounting guide so that the bracket base was parallel to the direction of the debonding force. Shear bond strength of the FRC-bracket interface was measured in an Instron Universal Testing Machine (Santam, model STM-20, Iran) using cross head speed of 1 mm/min. The force at failure point was recorded in newtons (N) and subsequently converted to megapascals (N/mm2) by dividing the force by the surface area of the bracket base (12.51 mm2).

The fracture sites were examined under a stereomicroscope at ×10 magnification to determine the amount of adhesive left on the tooth according to the adhesive remnant index (ARI) of Artun and Bergland (21): Score 0, no adhesive remained on the tooth; score1, less than 50% of the adhesive remained on the tooth; score 2, more than 50% of the adhesive remained on the tooth; score 3, the entire adhesive remained on the tooth with distinct impression of the bracket base.

Statistical analysis: The normal distribution of the data was confirmed by the Kolmogorov-Smirnov test and the homogeneity of variances by the Levene’s test. One-way analysis of variance (ANOVA) was run to determine any significant difference in bond strength among the study groups followed by Duncan post-hoc test for paier-wise comparisons. The difference in ARI scores among the six groups was assessed by Fisher’s exact test. The statistical analyses were performed using SPSS (Statistical Package for Social Sciences, Version 16.0, Chicago, IL, USA) software and the significance level was set at *P*<0.05.

## Results

 Table 1 presents the descriptive statistics and the results of statistical analyses for comparison of bond strength values among the study groups. The highest SBS (10.9 ± 0.95 MPa) was related to group 6 in which the thin layer of flowable composite on the FRC bar was treated by Er:YAG laser using 300 mJ of energy and frequency of 15 Hz. The lowest SBS belonged to group 1 (8.29 ± 0.84 MPa) in which neither the FRC surface was covered by flowable composite nor Er:YAG laser treatment was performed.

**Table 1:**
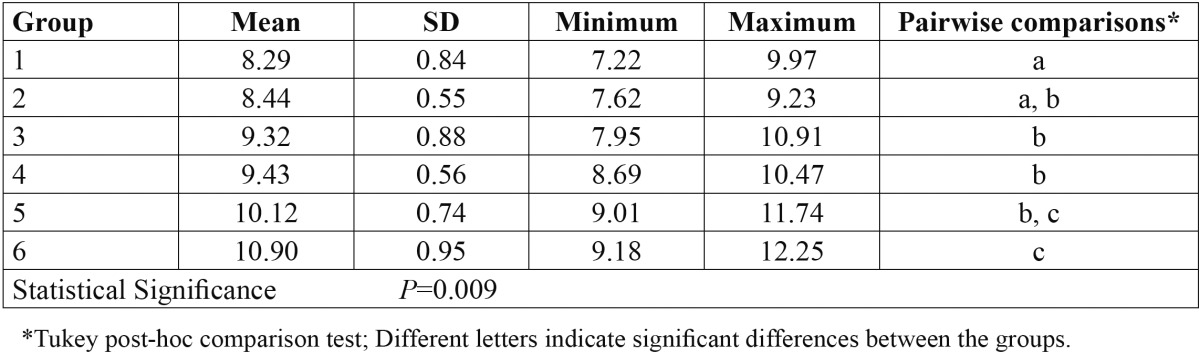
Descriptive statistics and the results of statistical analyses for comparison of bond strength values (MPa) among the study groups

ANOVA demonstrated a significant difference in bond strength values among the study groups (*P*=0.009;  Table 1). Pairwise comparisons by Duncan test revealed that SBS was significantly lower in group 1 compared to all other groups (*p*<0.05) except group 2 ( Table 1). Covering the FRC surface by a layer of flowable composite and then application of Er:YAG laser at 300 mJ/15 Hz (group 6) resulted in significantly higher bond strength compared to the other study groups (*p*<0.05) except group 5 in which 200 mJ/10 Hz was employed for surface treatment of resin composite over the FRC bar.

The ARI scores of the study groups are shown in  Table 2. In all groups, score 1 was dominant, indicating that failure site was mainly at the FRC-adhesive interface. ARI score 2, which represents failure site at the bracket-adhesive interface, was also prevalent in groups 5 and 6. Fisher’s exact test did not reveal any significant difference in the distribution of ARI scores among the various groups (*P*=0.15).

**Table 2:**
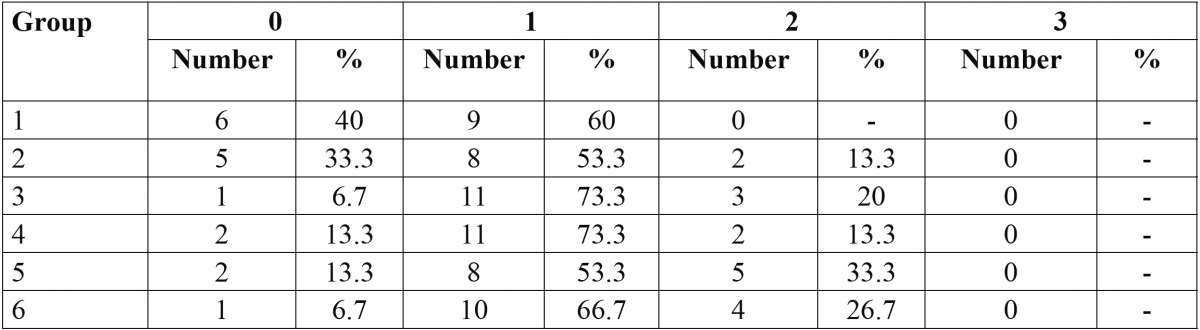
The distribution of adhesive remnant index (ARI) scores in the study groups.

## Discussion

Bond strength between attachments and FRCs should be high enough to resist intraoral forces during orthodontic treatment. The present study proved the efficacy of Er:YAG laser in surface conditioning of FRC bars as represented by the significant increase in bond strength of orthodontic brackets. A flowable composite was used for bonding FRCs to the teeth in this study, because of its higher viscosity compared to the light-cure or no-mix adhesive systems, which enables the operator to apply it in a thin layer. The use of flowable composite, however, is not suitable for bracket bonding because the attachments are inclined to drift until the resin is cured (22) and the resultant bond strength may be lower than that achieved by no-mix or light-cure systems (5,9).

In the present study, surface conditioning with Er:YAG laser was capable to significantly increase bond strength of brackets to FRC strips. Er:YAG laser was employed at 200 mJ/10 Hz or 300 mJ/15 Hz. Although the difference between the two laser groups was not significant, but group 6 in which the FRC surface was covered by a layer of flowable composite followed by application of Er:YAG laser at 300 mJ/15 Hz showed significantly higher bond strength compared to most of the study groups. Therefore, this protocol could be recommended to be employed in the clinical conditions to increase bond strength of orthodontic attachments to FRC strips.

Previous authors indicated the capability of Er:YAG laser in roughening different surfaces such as composite (15-16), amalgam (23) and bracket bases (17-18). Although microscopic evaluaion of the FRC surfaces was not performed in this study, it is possible that laser conditionng provides microporosities and microretentive areas on the FRC surface which increase the surface area for bonding, thus leading to enhanced bond strength of ortodontic attachments.

Adding a thin layer of flowable composite on the FRC bar before bracket bonding had no significant effect on SBS. However, when Er:YAG laser (300 mJ/15 Hz) was employed for conditioning the FRC surface covered by the flowable resin, a significant increase in bond strength was obtained compared to most of the study groups. In this case, Er:YAG laser actually roughened the composite surface instead of the FRC surface and brackets were bonded to the flowable composite. This increased bond strength compared to the groups in which Er:YAG laser was irradiated on FRC itself may be attributed to the even surface of the flowable composite that may be more appropriate for laser conditioning compared to the uneven FRC surface. Furthermore, it is possible that laser treatment causes some damage and weakens the FRC structure; thus reducing any positive effect of laser treatment on enhancing bond strength. The outcomes of this study are in agreement with those of previous authors who found that Er:YAG laser is effective in preparing the composite surfaces for the bonding process (15-16).

There are a few studies regarding bond strength of orthodontic attachments to FRC strips. Most of the previous studies evaluated the physical properties (1,8,24) or the effect of different adhesive systems on bond strength (5,9) of fiber-reinforced composite. Using hydroxyapatite stone instead of enamel, Freudenthaler *et al.* (4) evaluated bond strength of a metal attachment (control) with that of the FRC-attachment combination. They found that in all loading conditions, the FRC-attachment combination exhibited superior bond strength compared with the metal attachment alone, and under some loading conditions, the loads before failure were as much as 3 times greater than those for the control (4). In contrast, Sfondrini *et al.* (9) demonstrated that brackets bonded with FRC nets under the base showed significantly lower bond strength than those directly bonded to enamel (8.48 MPa versus 14.5 MPa using a light-cure adhesive). If we included a control group in the study design in which brackets were bonded to enamel, it was possible to compare SBS values of brackets bonded to FRC versus those attached to enamel.

In the present study, there was a higher frequency of ARI score 2 in groups 5 and 6 where greater SBS values were found, while ARI score 0 was more frequent in groups with lower SBS. A higher ARI score indicates that more adhesive remained on the tooth in the debonding area, representing fracture at the bracket-composite interface. The overall fracture pattern, however, was similar in all groups. The statistical analysis did not show any significant difference in debond locations among the six groups, possibly due to the small sample size or to the close range of SBS values observed in the present investigation.

According to Reynolds (25), bond strength of 6-8 MPa should be considered as the minimum strength required for orthodontic brackets to resist intraoral conditions. In the current study, all groups showed SBS values greater than the threshold proposed by Reynolds, although bond strength values were just slightly greater than 8 MPa in groups that laser conditioning was not performed. Surface treatment of FRC bars by adding a layer of flowable composite combined with Er:YAG laser conditioning enhanced SBS significantly and thus this technique can be recommended to be employed in the clinical practice.

## Conclusions

Under the conditions used in this study:

• Shear bond strength of orthodontic brackets to FRC bars were above the acceptable threshold required in clinical orthodontics.

• When maximum bond strength of orthodontic brackets is required, it is recommended to add a layer of flowable composite on FRC bar followed by surface treatment with Er:YAG laser.
